# A significant atypical U-shaped relationship exists between Lipid Accumulation Product and depression prevalence among Chinese middle-aged and elderly men: a cross-sectional study based on CHARLS

**DOI:** 10.3389/fnut.2025.1561990

**Published:** 2025-05-21

**Authors:** Chuanshen Gu, Xingzi Kang, Xinyi Chen, Zhengzheng Long, Fuxia Yang, Wenshu Luo

**Affiliations:** ^1^The Fourth Clinical Medical College of Guangzhou University of Chinese Medicine, Shenzhen, Guangdong, China; ^2^Shenzhen Traditional Chinese Medicine Hospital, Shenzhen, Guangdong, China; ^3^School of Ophthalmology, Chengdu University of Traditional Chinese Medicine, Chengdu, China; ^4^The First Affiliated Hospital of Guangzhou University of Chinese Medicine, Guangzhou University of Chinese Medicine, Guangzhou, Guangdong, China

**Keywords:** Lipid Accumulation Product (LAP), depression, China Health and Retirement Longitudinal Study (CHARLS), cross-sectional study, middle-aged and elderly Chinese men

## Abstract

**Background:**

The complex interplay of physiological conditions, chronic pathological changes, and social roles in middle-aged and elderly men presents significant challenges for clinicians in diagnosing depression within this group. Therefore, identifying simpler and more effective quantitative predictive indicators for depression risk is one of the urgent issues in the current medical system to prevent and treat depression in this population. Recent studies have found that fat accumulation has a bidirectional effect on mood, and that the Lipid Accumulation Product (LAP), a new indicator for assessing fat accumulation, may be associated with depression. However, there is no existing literature that explores the relationship between LAP and depressive symptoms in middle-aged and elderly men in China, nor any research comparing its predictive performance for depression risk against metabolic biomarkers.

**Methods:**

This study analyzed data from the 2015 and 2018 CHARLS surveys, with LAP divided into tertiles. Univariate logistic analysis and multivariable regression models were used to study the correlation between LAP and depressive symptoms. Subgroup analyses, interaction tests, and sensitivity analyses were conducted to validate the robustness of the model. Restricted cubic spline (RCS) regression was used to determine the potential threshold for LAP in relation to depression, revealing the non-linear relationship between LAP and depression. Finally, ROC curves were used to compare the predictive performance of LAP and metabolic biomarkers for depression risk.

**Results:**

Univariate logistic analysis and multivariable regression models explored the factors influencing depressive symptoms in middle-aged and elderly men in China, confirming the strong association and superior predictive performance of LAP for depression (*P* < 0.0001). RCS regression showed that, within a certain range, higher LAP levels significantly reduced depression risk in this population. Stratified subgroup analysis, interaction tests, and sensitivity analyses confirmed the stability of the results. ROC curves demonstrated that LAP had superior predictive performance for depression compared to traditional indicators and other metabolic biomarkers.

**Conclusion:**

This study applied more robust statistical methods to minimize the effects of confounding factors and identified a stable, atypical U-shaped relationship between LAP and the prevalence of depression in middle-aged and elderly men in China, as well as an effective threshold. The findings strongly support the “jolly fat” hypothesis in Chinese middle-aged and elderly men and offer guidance for dietary intake in this population.

## 1 Introduction

Depression ranks among the most prevalent mental health disorders affecting the elderly population globally, leading to considerable emotional distress and frequently coexisting with chronic illnesses and cognitive decline (1). This interplay exacerbates the depressive condition and contributes to elevated mortality rates (2). Presently, the rate of depression among middle-aged and older adults in China has escalated to 42% (3). Societal perceptions often depict men as symbols of strength and responsibility, tasked with the obligation to support their families. Nevertheless, the rapid advancement of modern society has corresponded with an annual rise in depression rates among men (4). In 2020, there was a 28% increase in the global number of individuals diagnosed with severe depression, with male cases rising by nearly 24%. Consequently, the evaluation and prediction of depression risk in middle-aged and elderly men have emerged as critical concerns for enhancing public health security (5). Previous studies have shown that depression is associated with various diseases, such as metabolic syndrome (6), diabetes (7), and chronic obstructive pulmonary disease (8). Notably, the pronounced correlation between depression and obesity warrants attention. Perspectives on the interplay between obesity and depression vary widely. Some researchers assert that obesity induces significant physiological, psychological, and behavioral alterations, which serve as potential risk factors for depression (9). They also propose that shared biological mechanisms underpin the relationship between obesity and depression, including the hypothalamic-pituitary-adrenal (HPA) axis, activation of immune inflammation, neuroendocrine modulation of energy metabolism, and the interplay between homeostatic and emotional regulatory circuits in the brain (10).Other researchers have introduced the “jolly fat hypothesis.” This theory suggests that moderate obesity might reduce the risk of depression, primarily through psychological factors like the pleasurable effects of a high-fat diet (11).

To evaluate the connection between obesity and overall health status, researchers have employed the Body Mass Index (BMI); however, it fails to differentiate between adipose and non-adipose tissues, nor does it account for the distribution of body fat, particularly abdominal fat (12). In 2005, Kahn introduced the Lipid Accumulation Product (LAP) index as a more effective indicator for assessing visceral fat accumulation and metabolic risk. The predictive performance of LAP was compared with that of BMI in regression models for risk variables such as HOMA-IR, fasting blood glucose, and A1C. The findings indicated that LAP provides a more accurate reflection of visceral fat accumulation and its associated biological toxicity, and outperforms BMI in predicting cardiovascular disease and diabetes (13). Studies have shown that LAP demonstrates superior efficacy compared to the Visceral Adiposity Index (VAI) in predicting certain lipid-related diseases (14).

LAP correlates with various diseases. Its straightforward calculation and cost-effectiveness make it a valuable tool for predicting disease risk and evaluating potential outcomes. A cross-sectional study utilizing data from NHANES 2017–2020 revealed a significant positive correlation between elevated LAP levels on the left side of the saturation point and an increased risk of osteoarthritis in adults (15). In addition, other studies have demonstrated that LAP provides high diagnostic accuracy for various metabolic syndromes (16, 17), and offers significant sensitivity and specificity in screening for non-alcoholic fatty liver disease (NAFLD) (18). However, due to the limitations of the calculation formula, this index is not suitable for assessing women with WC < 58 cm and men with WC < 65 cm, nor is it conducive to comparisons between different genders (19, 20). Given that visceral adipose tissue influences systemic metabolism through the release of free fatty acids (FFA) and pro-inflammatory factors such as IL-6 and TNF-α (21), and lipid toxicity due to excess fat accumulation is closely associated with insulin resistance and oxidative stress (22), which are mechanisms highly similar to those implicated in depression—namely, the activation of pro-inflammatory signaling pathways and depletion of antioxidant defenses leading to structural changes in the brain (23)—lipids play a crucial role in both intra-neuronal and interneuronal functions (24). Researchers posit that lipids regulate depression bidirectionally: they can maintain cerebral cortex and nerve activity and function; conversely, excessive lipid accumulation can induce chronic inflammation, with inflammatory factors crossing the blood-brain barrier to cause neuroinflammation and subsequent depressive symptoms (25). Socially and psychologically, obesity may damage self-esteem and lead to criticism, contributing to depression (26). As an indicator of lipid accumulation, LAP not only reflects the extent of lipid buildup but also sensitively captures early signals of metabolic disorders (27). The correlation between LAP and depression stems from the dual role of lipid accumulation, encompassing direct biological effects and interactions involving fatty acid metabolism, neuroinflammation, gender, age, and psychosocial factors. Therefore, LAP is anticipated to be an effective predictor of depression (28), and establishing its threshold could provide a quantitative standard for defining and distinguishing the bidirectional effects of lipids on depression, aiding clinical adjustments.

Although some studies have preliminarily explored the potential link between LAP and depression, there are few similar studies and they have not yet clearly explained the association between LAP and depression in Chinese middle-aged and elderly men. If a systematic investigation is conducted into the prevalence of depression among middle-aged and elderly men in China, and the potential significant association between LAP and depression is clarified, it will aid in the early identification of depression risk in this demographic.

Consequently, this study aims to investigate how LAP relates to the prevalence of depression in middle-aged and elderly men in China. We will clarify this relationship and emphasize LAP's significant potential as a new predictor for diagnosing and treating depression, compared to traditional measures like BMI and other metabolites.

## 2 Methods

### 2.1 Data and sample source

**Figure 1:**
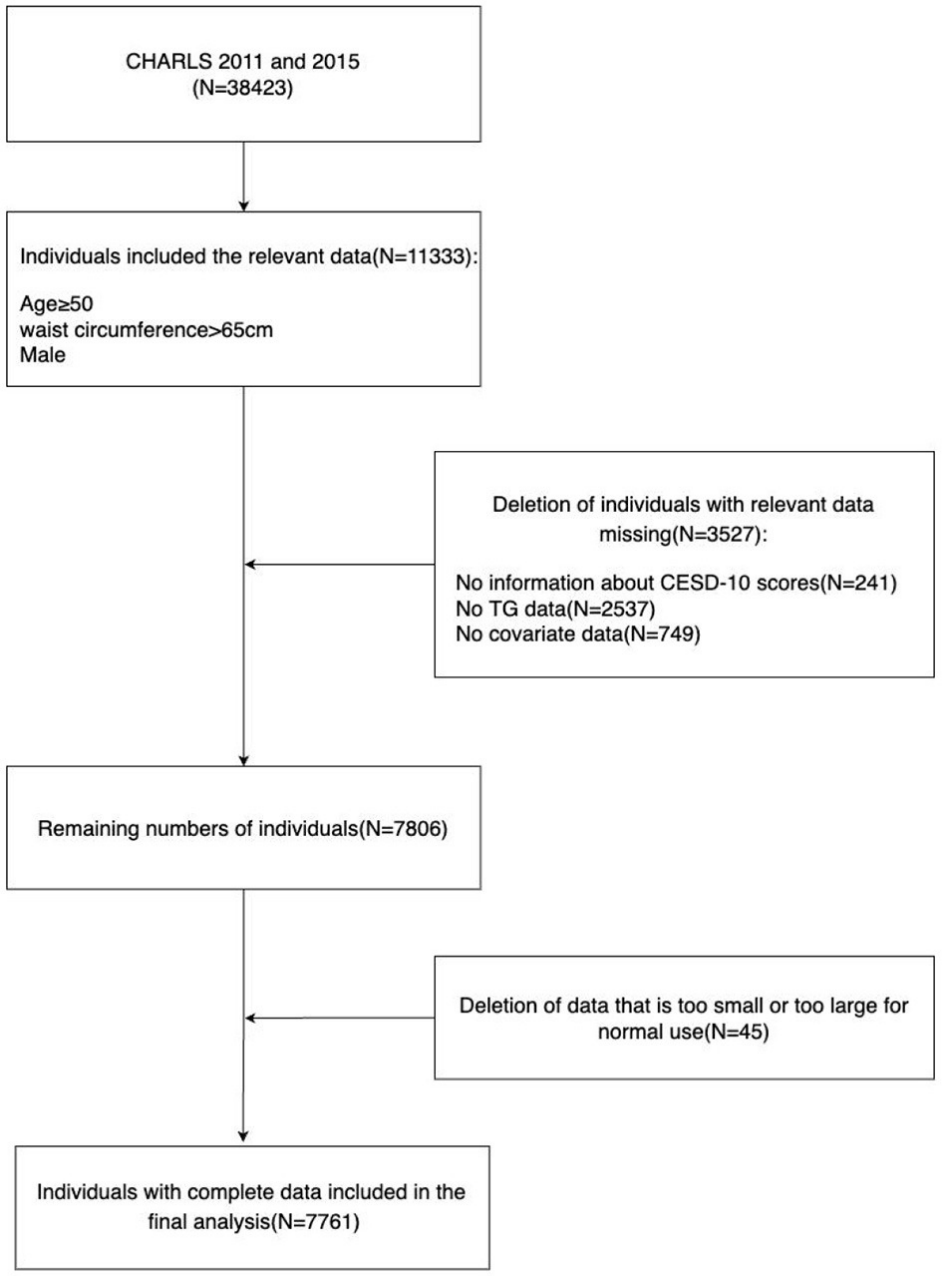
The flow chart of the included participants in this study.

CHARLS is a longitudinal survey of Chinese middle-aged and elderly people conducted by the National School of Development at Peking University with professional follow-up. The survey randomly selects individuals aged 45 and above as samples, and its respondents are distributed in both urban and rural areas, which can better represent the Chinese middle-aged and elderly population (29). Since 2011, the organizers have conducted a round of surveys every 2 years, and four rounds have been completed so far. All data used in this study are publicly provided by the National School of Development at Peking University, and researchers from all over the world can also access them (29) on the research website http://charls.pku.edu.cn/en. The study includes data from two CHARLS cycles (2011 and 2015), with a total of 38,423 participants. We selected 14,689 men who were at least 50 years old and had a waist circumference of at least 65 cm, excluding 241 people missing the 10-item Center for Epidemiologic Studies Depression Scale (CESD-10) scores, 2,537 people missing triglyceride data, 749 people missing covariate data, and 45 people with data that were too large or too small, obviously unreasonable, and finally included 7,761 male participants for analysis. The specific process of inclusion and exclusion is shown in Figure 1.

### 2.2 Ethics

CHARLS has been approved by the Ethics Committee of Peking University (IRB 00001052-11015), and written informed consent has been obtained from each subject before the survey.

### 2.3 Calculation of LAP

After initially introducing the concept of “LAP” in 2005, Kahn further refined the definition of LAP in 2006. Specifically, LAP was characterized as a theoretical measure of an individual's progression along the path of increasing waist circumference and triglyceride levels. Additionally, Kahn established sex-specific baselines using minimum waist circumference values and proposed distinct LAP calculation formulas for males and females. The formula for calculating male LAP is as follows: LA*P* = (WC – 65) × TG, where WC denotes waist circumference and TG denotes triglyceride level (13, 14).

### 2.4 Assessment of depressive symptoms

In 1994, Andresen et al. shortened the original 20-item Central Depression Scale used in epidemiological research to a 10-item version, aiming to evaluate respondents' feelings and behaviors over the past week. This reduction was intended to minimize response fatigue and better suit the assessment of depressive symptoms in elderly populations (30). The revised scale includes ten questions with four response options for each, reflecting the frequency of “negative,” “depressive,” “optimistic,” and “happy” emotions, ranging from “none or almost none” to “almost all of the time.” Each response is assigned a score from 0 to 3 based on the nature of the question, resulting in a total possible score between 0 and 30. According to the total score, depressive symptom severity is categorized as follows: no depressive symptoms (≤ 9), mild depressive symptoms (10–15), moderate depressive symptoms (16–25), and severe depressive symptoms (≥26). Subsequently, multiple studies have validated the psychometric properties of the CESD-10 in elderly populations, assessing its internal consistency, test-retest reliability, concurrent validity, and structural validity. The CESD-10 demonstrated satisfactory internal consistency (α = 0.78) and acceptable test-retest reliability (γ = 0.44) (31). Additionally, research evaluating the sensitivity and specificity of the CESD-10 found that using a cutoff score of 10 on the scale, which ranges from 0 to 30, yields high specificity in detecting depression among the elderly (32). In 2014, Chen et al., utilizing data from the CHARLS database, confirmed the factor validity of the CESD-10 for measuring mental health in middle-aged and elderly Chinese individuals. Their findings support the effectiveness, reliability, and utility of the CESD-10 in assessing depression among older adults in China, as well as its ability to distinguish gender differences in depressive symptomatology (33). Currently, the CESD-10, recognized for its robust reliability and validity, has been adopted by numerous developed countries as well as low-income developing countries, including China, to assess depressive symptoms (34–36). Consequently, this scale is extensively utilized in cross-sectional studies involving individuals with depression (37–42).

### 2.5 Covariates

Covariates include demographic information and health status and functional information. Demographic information includes age, marital status (unmarried and married), and education level (whether educated or not). Health status and functional information include current smoking, current alcohol consumption, exercise adherence, capacity to perform activities of daily living, height, weight, BMI, hypertension, diabetes, dyslipidemia, stroke, mental illness, and metabolic biomarkers. Metabolic biomarkers include total cholesterol (TC), triglycerides (TG), low-density lipoprotein cholesterol (LDL-C), and high-density lipoprotein cholesterol (HDL-C). The criteria for diagnosing hypertension (satisfying one of the following is sufficient): (1) Systolic blood pressure ≥140 mmHg; (2) Diastolic blood pressure ≥90 mmHg; (3) Currently using antihypertensive drugs; (4) Diagnosed with “hypertension”. The criteria for diagnosing diabetes (satisfying one of the following is sufficient): (1) Administration of insulin or oral hypoglycemic drugs; (2) Blood glucose level ≥200 mg/dL; (3) Diagnosed with “diabetes”. BMI is divided into three groups: underweight (BMI < 18.5 kg/m^2^), normal weight (18.5 kg/m^2^ ≤ BMI < 24 kg/m^2^), and overweight or obesity (BMI ≥ 24 kg/m^2^). The survey results of the 6-item Activities of Daily Living Scale (ADL) were utilized as the judging criteria. In this study, participants who were able to independently complete all six activities were categorized into the “0” group, whereas those who could not independently perform at least one of the six activities were classified into the “1” group.

### 2.6 Statistical analysis

Continuous variables are expressed as mean ± standard deviation (SD), and categorical variables are expressed as frequency and percentage. According to the type of data and its conformity to the normal distribution, Student's *t*-test, chi-square test, or Kruskal-Wallis test are used. Participants are divided into three groups based on the tertiles of LAP, and the baseline characteristics between the three groups are compared. The specific grouping is: T1 ( ≤ 33rd percentile), T2 (>33rd percentile, ≤ 66th percentile), and T3 (>66th percentile). When assessing the correlation between covariates other than metabolic biomarkers and depression, univariate logistic analysis is used; when assessing the correlation between LAP, metabolic biomarkers, and depression, a multivariate regression equation model is used. In addition, subgroup analysis and interaction tests are used to explore the relationship between LAP and depression in different subgroups to assess the stability and reliability of the study. Furthermore, regression equations were constructed for the subgroups with significant interaction, and regression coefficients and intercepts were calculated to evaluate the robustness of the relationship between LAP and depression. Subsequently, a restricted cubic spline curve (RCS) based on the multivariate logistic regression model is applied to investigate the non-linearity of the association between LAP and depression. If a non-linear relationship exists, a two-piece linear regression model is used to explain the differences in the association based on the threshold. The threshold refers to the threshold point with the highest possibility. Finally, the predictive performance of LAP for depression risk in middle-aged and elderly Chinese men was measured by receiver operating curve (ROC) and area under the curve (AUC), and compared with traditional indexes BMI and other metabolic markers. The log-likelihood ratio test is used to compare the association differences between the two-piece linear regression model and the single-line linear regression model. All analyses are conducted using R (version 4.2.1) and Empower Stats (version 4.2). Statistical significance is determined based on a two-sided *P* < 0.05.

## 3 Results

### 3.1 Basic characteristics of the participant population

The basic characteristics of the participants were analyzed according to the tertiles of LAP (Table 1). In terms of demographic information, participants with higher LAP were younger on average (T1:64.04 ± 8.50; T2:63.43 ± 8.23; T3:61.40 ± 7.74), mostly married (T1:87.74%; T2:88.95%; T3:92.50%), and had received at least basic education (T1:58.68%; T2:66.32%; T3:74.14%). In terms of functional information, participants with higher height (T1:1.62 ± 0.08; T2:1.63 ± 0.08; T3:1.65 ± 0.07), heavier weight (T1:53.46 ± 7.27; T2:61.93 ± 8.71; T3:71.26 ± 10.64), overweight as indicated by BMI (T1:4.56%; T2:33.10%; T3:72.17%), and higher levels of TC (T1:173.36 ± 33.36; T2:180.80 ± 34.91; T3:191.34 ± 38.04), TG (T1:74.34 ± 28.85; T2:103.96 ± 36.58; T3:209.58 ± 114.79), and LDL-C (T1:99.61 ± 29.19; T2:108.33 ± 30.75; T3:106.55 ± 34.57) were more likely to have higher LAP, while their HDL-C (T1:57.90 ± 15.16; T2:51.04 ± 12.85; T3:42.77 ± 10.08) levels were likely to be lower. In terms of health status, participants with higher LAP were more likely to have hypertension (T1:39.26%; T2:51.95%; T3:64.48%), diabetes (T1:10.41%; T2:13.36%; T3:26.63%), dyslipidemia (T1:6.65%; T2:12.01%; T3:25.63%), and stroke (T1:2.79%; T2:3.40%; T3:4.83%), and the above results had statistically significant differences (*P* < 0.001).Additionally, participants who were currently smoking had lower LAP, but no significant differences were observed in current alcohol consumption, exercise adherence, capacity to perform activities of daily living, height, or mental illness (*P* > 0.05). As shown in Table 2, participants with depressive symptoms were classified as having mild, moderate, or severe depressive symptoms according to the CESD-10 scoring criteria, and their basic characteristics were analyzed based on the tertiles of LAP to observe the relationship between LAP and the severity of depressive symptoms. Furthermore, participants who had at least one of the two diseases were distinguished as having only stroke, only depressive symptoms, or comorbid stroke and depressive symptoms to analyze the association between LAP and these two diseases. The results indicate that among participants showing depressive symptoms, those with higher LAP had milder depressive symptoms, and most participants with higher LAP had a lower likelihood of depression, but the prevalence of comorbid stroke and stroke-related depression was higher with statistically significant differences (*P* < 0.001).

**Table 1:** Characteristics of participants in the CHARLS 2011 and 2015 (*n* = 7,761).

**Characteristics**	**lipid accumulation product (LAP) (*****N** =* **7,761)**			***P*-value**
	**T1 (*****N** =* **2585)**	**T2 (*****N** =* **2589)**	**T3 (*****N** =* **2587)**	
Age, years	64.04 ± 8.50	63.43 ± 8.23	61.40 ± 7.74	< 0.001
Married, %	2,268 (87.74%)	2,303 (88.95%)	2,393 (92.50%)	< 0.001
Educated, %	1,517 (58.68%)	1,717 (66.32%)	1,918 (74.14%)	< 0.001
Smoking, %	1,625 (62.86%)	1,421 (54.89%)	1,226 (47.39%)	< 0.001
Drinking, %	1,413 (54.66%)	1,437 (55.50%)	1,460 (56.44%)	0.438
Exercise, %	1,068 (90.20%)	1,099 (90.68%)	1,087 (89.91%)	0.813
ADL6, %	417 (16.13%)	440 (16.99%)	433 (16.74%)	0.693
Height, m	1.62 ± 0.08	1.63 ± 0.08	1.65 ± 0.07	< 0.001
Weight, kg	53.46 ± 7.27	61.93 ± 8.71	71.26 ± 10.64	< 0.001
BMI, kg/m^2^	20.60 ± 7.59	23.46 ± 10.91	26.44 ± 17.02	< 0.001
**BMI, kg/m**^2^ **(Tertile)**				< 0.001
< 18.5	437 (16.91%)	50 (1.93%)	8 (0.31%)	
18.5 to < 24	2,030 (78.53%)	1,682 (64.97%)	712 (27.52%)	
≥24	118 (4.56%)	857 (33.10%)	1,867 (72.17%)	
HTN, %	1,015 (39.26%)	1,345 (51.95%)	1,668 (64.48%)	< 0.001
DM, %	269 (10.41%)	346 (13.36%)	689 (26.63%)	< 0.001
Dyslipidemia, %	172 (6.65%)	311 (12.01%)	663 (25.63%)	< 0.001
Stroke, %	72 (2.79%)	88 (3.40%)	125 (4.83%)	< 0.001
Psychosis, %	37 (1.43%)	32 (1.24%)	28 (1.08%)	0.527
**Metabolic biomarkers**				
Total cholesterol, mg/dl	173.36 ± 33.36	180.80 ± 34.91	191.34 ± 38.04	< 0.001
Triglycerides, mg/dl	74.34 ± 28.85	103.96 ± 36.58	209.58 ± 114.79	< 0.001
LDL cholesterol, mg/dl	99.61 ± 29.19	108.33 ± 30.75	106.55 ± 34.57	< 0.001
HDL cholesterol, mg/dl	57.90 ± 15.16	51.04 ± 12.85	42.77 ± 10.08	< 0.001

T, Tertile; LDL, Low-Density Lipoprotein Cholesterol; HDL, High-Density Lipoprotein Cholesterol; DM, Diabetes Mellitus; HTN, Hypertension; BMI, Body Mass Index; ADL6, Activities of Daily Living Scale.

**Table 2:** Baseline prevalence of depressive symptoms among participants.

**Depressive symptoms (*n*, %)**	**Lipid accumulation product (LAP) (*****N** =* **7,761)**			***P*-value**
	**T1 (*****N** =* **2,585)**	**T2 (*****N** =* **2,589)**	**T3 (*****N** =* **2,587)**	
Overall depressive symptom	882 (34.12%)	713 (27.54%)	637 (24.62%)	< 0.001
Mild depressive symptom	476 (18.41%)	409 (15.80%)	382 (14.77%)	< 0.001
Medium depressive symptom	372 (14.39%)	272 (10.51%)	231 (8.93%)	< 0.001
Severe depressive symptom	34 (1.32%)	32 (1.24%)	24 (0.93%)	< 0.001
Stroke only	29 (1.12%)	55 (2.12%)	68 (2.63%)	< 0.001
Depressive symptoms only	839 (32.46%)	680 (26.26%)	580 (22.42%)	< 0.001
Stroke and Depressive symptoms	43 (1.66%)	33 (1.27%)	57 (2.20%)	< 0.001

### 3.2 Univariate regression analysis of covariates and depression

As shown in Table 3, univariate regression analysis was used to observe the association between age, marital status, education level, current smoking, current alcohol consumption, height, weight, BMI, hypertension, diabetes, dyslipidemia, stroke, mental illness, and depression. In terms of age, for every 1-year increase, the prevalence of depression among participants will rise by 1% (OR 1.01, 95% CI: 1.00–1.02, *P* = 0.0005). In terms of marital status, compared with unmarried individuals, those who are married are less likely to suffer from depression (OR 0.53, 95% CI: 0.46–0.62, *P* < 0.0001). In terms of education level, individuals who have received education are less likely to suffer from depression than those who have not (OR 0.61, 95% CI: 0.55–0.68, *P* < 0.0001). In terms of lifestyle habits, smokers are more likely to suffer from depression than those who have never smoked or have quit smoking (OR 1.18, 95% CI: 1.06–1.30, *P* = 0.0014); compared with those who never drink or have quit drinking, drinkers have a lower proportion of depression (OR 0.77, 95% CI: 0.70–0.85, *P* < 0.0001). In terms of height, taller individuals have a significantly reduced likelihood of suffering from depression (OR 0.17, 95% CI: 0.09–0.33, *P* < 0.0001). In terms of weight, an increase in weight may reduce the prevalence of depression (OR 0.98, 95% CI: 0.97–0.98, *P* < 0.0001). In terms of BMI, as BMI increases, the probability of participants suffering from depression decreases, and individuals with a BMI of 24 or above have a lower prevalence of depression (OR 0.49, 95% CI: 0.40–0.60, *P* < 0.0001) compared to those with a BMI between 18.5 and 24 (OR 0.71, 95% CI: 0.59–0.86, *P* = 0.0005). In terms of health status, individuals with dyslipidemia are more likely to suffer from depression than those without dyslipidemia (OR 1.15, 95% CI: 1.01–1.32, *P* = 0.0377). Individuals with stroke (OR 2.24, 95% CI: 1.77–2.84, *P* < 0.0001), individuals with mental illness (OR 3.03, 95% CI: 2.03–4.54, *P* < 0.0001) and individuals with limited ability to perform activities of daily living (OR 3.90, 95% CI: 3.45–4.42, *P* < 0.0001) have a significantly increased prevalence of depression.

**Table 3:** Univariate analysis of the association between depression with covariates.

**Variable**	**OR (95 % CI)**	***P*-value**
Age	1.01(1.00, 1.02)	0.0005
**Married**		
No	Ref	–
Yes	0.53 (0.46, 0.62)	< 0.0001
**Educated**		
No	Ref	–
Yes	0.61 (0.55, 0.68)	< 0.0001
**Smoking**		
No	Ref	–
Yes	1.18 (1.06, 1.30)	0.0014
**Drinking**		
No	Ref	–
Yes	0.77 (0.70, 0.85)	< 0.0001
**Exercise**		
No	Ref	–
Yes	0.87 (0.68, 1.10)	0.2467
**ADL6**		
No	Ref	–
Yes	3.90 (3.45, 4.42)	< 0.0001
Height	0.17 (0.09, 0.33)	< 0.0001
Weight	0.98 (0.97, 0.98)	< 0.0001
BMI	0.97 (0.96, 0.98)	< 0.0001
**BMI (Tertile)**		
< 18.5	Ref	–
≥18.5, < 24	0.71 (0.59, 0.86)	0.0005
≥24	0.49 (0.40, 0.60)	< 0.0001
**HTN**		
No	Ref	–
Yes	1.04 (0.94, 1.15)	0.4053
**DM**		
No	Ref	–
Yes	1.11 (0.98, 1.27)	0.1078
**Dyslipidemia**		
No	Ref	–
Yes	1.15 (1.01, 1.32)	0.0377
**Stroke**		
No	Ref	–
Yes	2.24 (1.77, 2.84)	< 0.0001
**Psychosis**		
No	Ref	–
Yes	3.03 (2.03, 4.54)	< 0.0001

DM, Diabetes Mellitus; HTN, Hypertension; BMI, Body Mass Index; CI, confidence interval; OR, odds ratio.ADL6, Activities of Daily Living Scale.

### 3.3 Multivariate regression equations for LAP, metabolic biomarkers, and depression

As shown in Table 4, three multivariate logistic regression equation models were established to analyze the relationship between LAP, metabolic biomarkers, and depression: Model 1 was unadjusted, Model 2 was adjusted for selected covariates (age, marital status, education level, current smoking, current alcohol consumption, and exercise adherence, capacity to perform activities of daily living), and Model 3 was adjusted for all covariates except height, weight, and metabolic biomarkers (age, marital status, education level, BMI, current smoking, current alcohol consumption, exercise adherence, capacity to perform activities of daily living, stroke, mental illness, dyslipidemia, hypertension, and diabetes). In all three models, participants with medium (model 1:OR 0.73, 95%CI: 0.65–0.83, *P* < 0.0001; model 2:OR 0.73, 95%CI: 0.61–0.88, *P* = 0.0009; model 3:OR 0.73, 95% CI: 0.60–0.88, *P* = 0.001) and high LAP (model 1:OR 0.63, 95% CI: 0.56–0.71, *P* < 0.0001; model 2:OR 0.65, 95%CI: 0.54–0.79, *P* < 0.0001; model 3:OR 0.61, 95% CI: 0.49–0.76, *P* < 0.0001) had significantly reduced prevalence of depression, and the prevalence of depression among participants with high LAP was lower than those with medium LAP. Additionally, participants with high TG had a lower prevalence of depression, but those with high HDL-C had a significantly higher prevalence of depression (model 1:OR 1.21, 95%CI: 1.08–1.37, *P* = 0.0017; model 2:OR 1.32, 95% CI: 1.09–1.59, *P* = 0.0048; model 3:OR 1.30, 95%CI: 1.06–1.59, *P* = 0.0105). The remaining metabolic biomarkers had no statistically significant impact on the prevalence of depression among participants (*P* > 0.05).

**Table 4:** Multivariate regression equations for LAP, metabolic biomarkers, and depression.

**Exposure**	**Tertile group**	**Model 1**		**Model 2**		**Model 3**	
		**OR (95% CI)**	***P*** **-value**	**OR (95% CI)**	***P*** **-value**	**OR (95% CI)**	***P*** **-value**
LAP	Low	Ref	–	Ref	–	Ref	–
	Middle	0.73 (0.65, 0.83)	< 0.0001	0.73 (0.61, 0.88)	0.0009	0.73 (0.60, 0.88)	0.0010
	High	0.63 (0.56, 0.71)	< 0.0001	0.65 (0.54, 0.79)	< 0.0001	0.61 (0.49, 0.76)	< 0.0001
TC	Low	Ref	–	Ref	–	Ref	–
	Middle	0.94 (0.84, 1.06)	0.3296	0.97 (0.80, 1.16)	0.7271	0.97 (0.81, 1.17)	0.7691
	High	0.92 (0.81, 1.04)	0.1665	0.91 (0.75, 1.09)	0.3055	0.90 (0.74, 1.09)	0.2638
TG	Low	Ref	–	Ref	–	Ref	–
	Middle	0.93 (0.83, 1.05)	0.2365	0.96 (0.80, 1.15)	0.6407	0.98 (0.81, 1.18)	0.7952
	High	0.75 (0.66, 0.85)	< 0.0001	0.83 (0.68, 1.00)	0.0511	0.83 (0.68, 1.01)	0.0691
LDL	Low	Ref	–	Ref	–	Ref	–
	Middle	0.99 (0.88, 1.11)	0.8432	1.00 (0.84, 1.20)	0.9732	1.02 (0.85, 1.22)	0.8647
	High	0.90 (0.79, 1.01)	0.0761	0.79 (0.66, 0.96)	0.0179	0.80 (0.66, 0.97)	0.0221
HDL	Low	Ref	–	Ref	–	Ref	–
	Middle	0.97 (0.86, 1.10)	0.6781	0.99 (0.82, 1.20)	0.8977	0.97 (0.80, 1.18)	0.7780
	High	1.21 (1.08, 1.37)	0.0017	1.32 (1.09, 1.59)	0.0048	1.30 (1.06, 1.59)	0.0105

TC, Total Cholesterol; TG, Triglycerides; LDL, Low-Density Lipoprotein Cholesterol; HDL, High-Density Lipoprotein Cholesterol; CI, confidence interval; OR, odds ratio.

Model 1: Non-adjusted.

Model 2: adjust for Age; Married; Educated; Smoking; Drinking; Exercise; ADL6.

Model 3: adjust for Age; Married; Educated; Smoking; Drinking; Exercise; ADL6; BMI; HTN; DM; Dyslipidemia; Stroke; Psychosis.

### 3.4 Subgroup analysis interaction test and sensitivity analysis

As shown in Table 5: To determine the stability of the correlation between LAP and the prevalence of depression among participants, we conducted subgroup analysis and interaction tests, excluding TG from the subgroup analysis and interaction tests due to its potential impact on the results. The results showed that among married participants (T2: OR 0.70, 95%CI: 0.62–0.80, P < 0.0001; T3: OR 0.62, 95%CI: 0.54–0.71, *P* < 0.0001) and those without dyslipidemia (T2: OR 0.71, 95%CI: 0.63–0.81, *P* < 0.0001; T3: OR 0.62, 95%CI: 0.55–0.71, *P* < 0.0001), those with higher LAP had a lower prevalence of depression; among individuals with dyslipidemia, those with higher LAP had a significantly decreased prevalence of depression (T2: OR 0.74, 95%CI: 0.51–1.09, *P* = 0.1255; T3: OR 0.49, 95%CI: 0.35–0.69, *P* < 0.0001). Whether smokers (T2: OR 0.75, 95% CI: 0.64–0.87, *P* = 0.0002; T3: OR 0.62, 95% CI: 0.52–0.73, *P* < 0.0001), ex-smokers, or never smokers (T2: OR 0.73, 95%CI: 0.61–0.89, *P* = 0.0013; T3: OR 0.67, 95%CI: 0.56–0.81, *P* < 0.0001), or those with limited daily activity function (T2: OR 0.63, 95%CI: 0.48–0.84, *P* = 0.0019; T3: OR 0.45, 95%CI: 0.32–0.62, *P* < 0.0001) and normal daily activity function (T2: OR 0.76, 95%CI: 0.66–0.88, *P* = 0.0002; T3: OR 0.68, 95%CI: 0.88–0.80, *P* < 0.0001), a higher LAP meant a lower prevalence of depression. Notably, the reduction effect of LAP on depression risk was more pronounced in individuals with limited daily activities. Additionally, in individuals without psychiatric disorders, the prevalence of depression decreased as LAP increased (T2: OR 0.74, 95% CI: 0.66–0.83, *P* < 0.0001; T3: OR 0.63, 95% CI: 0.56–0.71, *P* < 0.0001). No statistically significant differences were observed in other subgroups in the interaction test (*P* > 0.05). This indicates that the negative correlation between LAP and the prevalence of depression among participants is stable in most subgroups, supporting the reliability of the study. In addition, psychiatric disorders and dyslipidemia may serve as confounding factors that influence the association between LAP and the prevalence of depression among participants. To further investigate this relationship, we fitted regression models between LAP and depression for several important subgroups (age, smoking status, marital status, dyslipidemia, and mental illness), measuring the regression coefficients and intercepts to assess the stability of these associations across different subgroups. As shown in Table 6, the relationship between LAP and depression is negatively correlated across different age groups, but this correlation is weaker in the 50–58 age group (regression coefficient = −0.00287). In contrast, the impact of LAP on depression appears more pronounced in the 59–65 (regression coefficient = −0.00507) and 66–94 (regression coefficient = −0.00428) age groups. For both smokers and non-smokers, there is a significant negative correlation between LAP and depression, with the effect being stronger in smokers (regression coefficient = −0.00540) compared to non-smokers (regression coefficient = −0.00261). Additionally, the married group exhibits a slightly higher regression coefficient (−0.00383) compared to the unmarried group (−0.00295), suggesting that marriage may provide some protective effect against depression in the context of LAP. In subgroups with and without hyperlipidemia, a negative correlation persists, but it is slightly stronger in those without hyperlipidemia (regression coefficient = −0.00512) compared to those with hyperlipidemia (regression coefficient = −0.00440), indicating that hyperlipidemia may attenuate the relationship between LAP and depression. There is a significant difference in the relationship between LAP and depression between the groups with and without psychiatric disorders. The regression coefficient for the group without psychiatric disorders (−0.00455) is markedly different from that of the group with psychiatric disorders (0.00545). In patients with psychiatric disorders, there is a positive correlation between LAP and depression. In contrast, in individuals without psychiatric disorders, LAP and depression remain negatively correlated, suggesting that the presence of psychiatric disorders may alter the relationship between LAP and depression, leading to a positive correlation. Overall, although there are differences in the magnitude of the regression coefficients, the relationship between LAP and depression remains largely negative across different subgroups, indicating that the effect of LAP on depression is stable in these subgroups and the overall trend is consistent.

**Table 5:** Hierarchical analysis and interaction effects.

**Variables^a^**	**Lipid accumulation product (LAP)**					***P* for interaction**
	**TI**	**T2**	***P*** **-value**	**T3**	***P*** **-value**	
**Age (Tertile)**						0.1509
50–58	Ref	0.68 (0.55, 0.86)	0.0009	0.64 (0.52, 0.80)	< 0.0001	
59–65	Ref	0.79 (0.64, 0.98)	0.0282	0.59 (0.48, 0.73)	< 0.0001	
66–94	Ref	0.73 (0.61, 0.88)	0.0012	0.69 (0.56, 0.85)	0.0004	
**Married**						0.0264
No	Ref	1.03 (0.74, 1.42)	0.8683	0.90 (0.63, 1.30)	0.5821	
Yes	Ref	0.70 (0.62, 0.80)	< 0.0001	0.62 (0.54, 0.71)	< 0.0001	
**Smoking**						0.0061
No	Ref	0.73 (0.61, 0.89)	0.0013	0.67 (0.56, 0.81)	< 0.0001	
Yes	Ref	0.75 (0.64, 0.87)	0.0002	0.62 (0.52, 0.73)	< 0.0001	
**Drinking**						0.1325
No	Ref	0.73 (0.61, 0.87)	0.0004	0.67 (0.56, 0.80)	< 0.0001	
Yes	Ref	0.74 (0.63, 0.87)	0.0003	0.60 (0.51, 0.71)	< 0.0001	
**Exercise**						0.9375
No	Ref	0.78 (0.41, 1.51)	0.4664	0.55 (0.24, 1.27)	0.1615	
Yes	Ref	0.77 (0.64, 0.94)	0.0087	0.70 (0.57, 0.87)	0.0014	
**ADL6**						0.0165
No	Ref	0.76 (0.66, 0.88)	0.0002	0.68 (0.58, 0.80)	< 0.0001	
Yes	Ref	0.63 (0.48, 0.84)	0.0019	0.45 (0.32, 0.62)	< 0.0001	
**BMI (Tertile)**						0.1414
< 18.5	Ref	0.60 (0.31, 1.14)	0.1204	2.57(0.61, 10.88)	0.2006	
≥18.5, < 24	Ref	0.87 (0.76, 1.00)	0.0559	0.76 (0.63, 0.92)	0.0048	
≥24	Ref	0.55 (0.36, 0.83)	0.0043	0.58 (0.39, 0.85)	0.0060	
**HTN**						0.4227
No	Ref	0.74 (0.62, 0.87)	0.0002	0.57 (0.47, 0.69)	< 0.0001	
Yes	Ref	0.71(0.60,0.85)	0.0002	0.64 (0.54, 0.75)	< 0.0001	
**DM**						0.3975
No	Ref	0.75 (0.66, 0.85)	< 0.0001	0.60 (0.53, 0.69)	< 0.0001	
Yes	Ref	0.61 (0.43, 0.85)	0.0039	0.61 (0.45, 0.82)	0.0009	
**Dyslipidemia**						0.0055
No	Ref	0.71 (0.63, 0.81)	< 0.0001	0.62 (0.55, 0.71)	< 0.0001	
Yes	Ref	0.74 (0.51, 1.09)	0.1255	0.49 (0.35, 0.69)	< 0.0001	
**Stroke**						0.6509
No	Ref	0.75 (0.66, 0.84)	< 0.0001	0.61 (0.54, 0.70)	< 0.0001	
Yes	Ref	0.40 (0.21, 0.77)	0.0055	0.57 (0.31, 1.02)	0.0573	
**Psychosis**						0.0038
No	Ref	0.74 (0.66, 0.83)	< 0.0001	0.63 (0.56, 0.71)	< 0.0001	
Yes	Ref	0.53 (0.20, 1.38)	0.1946	1.05 (0.39, 2.87)	0.9186	
**TC**						0.6802
Low	Ref	0.88 (0.73, 1.07)	0.2084	0.71 (0.57, 0.89)	0.0024	
Middle	Ref	0.64 (0.52, 0.78)	< 0.0001	0.49 (0.40, 0.61)	< 0.0001	
High	Ref	0.68 (0.55, 0.86)	0.0010	0.69 (0.56, 0.86)	0.0007	
**LDL (Tertile)**						0.7340
Low	Ref	0.78 (0.63, 0.96)	0.0168	0.61 (0.49, 0.74)	< 0.0001	
Middle	Ref	0.81 (0.66, 0.98)	0.0344	0.65 (0.52, 0.80)	< 0.0001	
High	Ref	0.64 (0.52, 0.80)	< 0.0001	0.64 (0.52, 0.79)	< 0.0001	
**HDL (Tertile)**						0.6665
Low	Ref	0.66 (0.50, 0.86)	0.0020	0.63 (0.49, 0.80)	0.0002	
Middle	Ref	0.82 (0.67, 1.01)	0.0563	0.62 (0.49, 0.77)	< 0.0001	
High	Ref	0.73 (0.61, 0.88)	0.0008	0.63 (0.47, 0.83	0.0013	

T: Tertile, LDL: Low-Density Lipoprotein Cholesterol; HDL: High-Density Lipoprotein Cholesterol; DM: Diabetes Mellitus; HTN: Hypertension; BMI: Body Mass Index; TC: Total Cholesterol.ADL6:Activities of Daily Living Scale.

^a^All variables were adjusted for each element and analyzed.

**Table 6:** Sensitivity analysis result.

**Variables**	**Coefficient**	**Intercept**
**Married**		
No	−0.00295	−0.2759
Yes	−0.00383	−0.8507
**Smoking**		
No	−0.00261	−0.9017
Yes	−0.00540	−0.6782
**Dyslipidemia**		
No	−0.00512	−0.7813
Yes	−0.00440	−0.5522
**Psychosis**		
No	−0.00455	−0.7761
Yes	0.00545	−0.0102

Coefficient: regression coefficient.

### 3.5. RCS curve of multivariate logistic regression model

**Figure 2:**
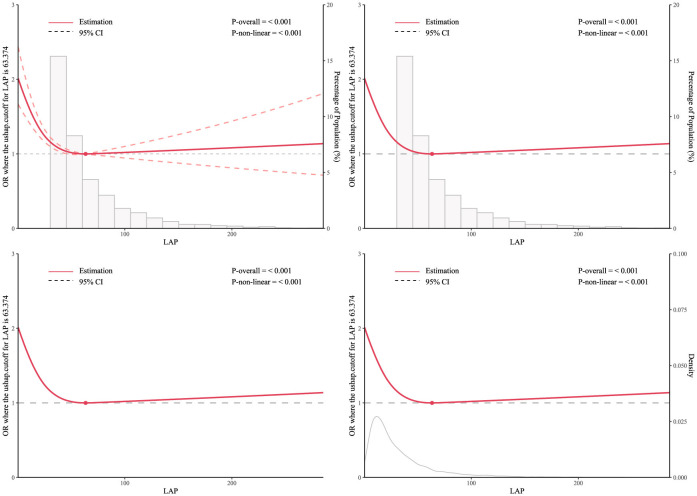
RCS curve model of the association between LAP and depression prevalence among middle-aged and elderly Chinese men.

As shown in Figure 2: The RCS curve was used to further explore the negative correlation between LAP and the prevalence of depression among participants, adjusting for all covariates in the model. The results showed a non-linear relationship between LAP and the prevalence of depression among participants. When LAP was >0 and < 63.374, the prevalence of depression among participants decreased with the increase of LAP; when LAP was >63.374, the prevalence of depression among participants slowly increased with the increase of LAP.

### 3.6 Subject operating curve measurement

**Figure 3:**
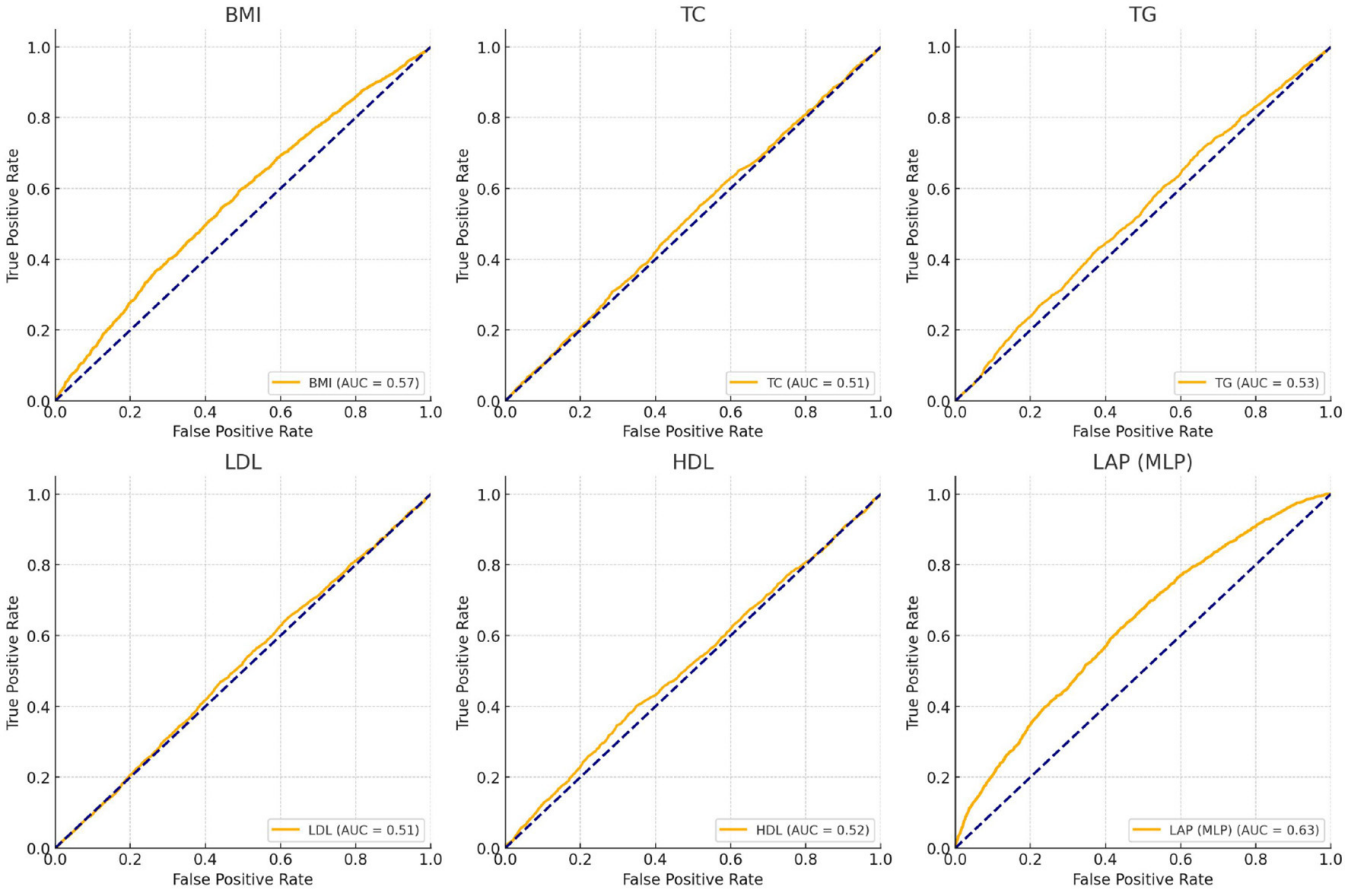
ROC curves for predicting depression using LAP, BMI, and metabolic biomarkers.

As shown in Figure 3, the effectiveness of LAP, BMI, and other metabolic markers in predicting depression risk was assessed by using ROC curves and AUC values. The results showed that the LAP model (AUC = 0.63) trained by the neural network (MLP) predicted depression better than the BMI model (AUC = 0.57), while the AUC values of other metabolic marker models were generally close to 0.50, suggesting that these features were weak in predicting depression.

## 4 Discussion

This study analyzed data from male individuals over 50 years old in the 2015 and 2018 CHARLS to investigate the association between LAP and the prevalence of depression among Chinese middle-aged and elderly men, as well as the influencing factors of depression prevalence in this population. The distribution of the severity of depressive symptoms among participants in the LAP tertiles indicated that individuals with higher LAP had milder depressive symptoms, and the proportion of individuals with varying degrees of depressive symptoms decreased with increasing LAP. Furthermore, we observed the characteristics of participant populations with stroke only, depression only, and comorbid stroke and depression within the LAP tertiles. The results found that with the increase of LAP, the prevalence of participants with pure stroke and comorbid stroke and depression increased, while the prevalence of pure depression decreased. Univariate analysis indicated that age, marital status, education level, current smoking and drinking, limitations in activities of daily living, height, weight, BMI, dyslipidemia, stroke, and mental illness are all influencing factors of depression prevalence. Multiple multivariate regression equations showed that the impact of LAP tertiles on the prevalence of depression among participants was statistically significant (*P* < 0.05), but among pure metabolic biomarkers, only high levels of TG and HDL-C had a statistically significant impact on the prevalence of depression among participants. In addition, subgroup analysis and interaction tests showed that the stability of the relationship between LAP and the prevalence of depression among participants was not affected by most stratification factors, and sensitivity analysis results suggested that the negative correlation between LAP and the prevalence of depression in participants persisted in most subgroups. Then, the dose-response relationship between LAP and depression risk was further examined using the RCS curve. A turning point of 63.374 on the U-shaped curve was identified, indicating a significant change in the relationship between LAP and depression risk at this threshold. In conclusion, the dose-response association between these variables was investigated using the RCS curve analysis. The findings indicated a notable atypical U-shaped relationship between LAP and the incidence of depression in Chinese men of middle and older age. Specifically, a moderate level of lipid accumulation appears to be associated with a decreased prevalence of depression in this demographic; conversely, excessive lipid accumulation is linked to an increased incidence of depression within the same population. Finally, the ROC curve and AUC values indicated that LAP, after MLP training, outperformed traditional BMI and other metabolites in predicting depression.

Contemporary theories indicate a clear association between the incidence of depression and lipid dysregulation. Lipid metrics influence cognitive function not only through direct biological mechanisms but may also exert an indirect impact via psychological factors, including depression, thus affecting mood and mental health (43). However, the relationship between lipids and depression is often contradictory in existing research results. For example, on the one hand, the intake of high-fat diets may promote the occurrence of anxiety and depressive behaviors by affecting gut microbiota and neuroinflammation (44), on the other hand, some unsaturated fatty acids can improve depressive symptoms (45). These contradictions may be related to factors such as the measure of lipid dysregulation, the direction of regulation, the type of lipid and the amount of fat accumulation.

As an emerging indicator to measure visceral fat, the LAP not only reflects the extent of fat accumulation but also indicates the toxic effects of lipids. Consequently, LAP has been widely utilized in predicting and preventing various diseases, including stress incontinence (46) and type 2 diabetes (47). Studies have shown that there may be a correlation between LAP and depression. Zhu et al. conducted a cross-sectional study using NHANES data from 2005 to 2018, revealing a positive correlation between LAP and depression (48). Similarly, Dai et al. performed another cross-sectional study in the same year, corroborating these findings. Specifically, individuals with higher LAP levels may be at increased risk for depression, particularly among men and those with hypertension (49). This view was also supported by Kong et al. in 2025, who found that when LAP is < 140.16 cm × mmol/L, it serves as an independent risk factor for depression among American adults, particularly significant in women and never-smokers (50). However, these studies are based on cross-sectional analyses of the NHANES database in the United States, which do not separately examine the male population or include Chinese middle-aged and elderly men. Therefore, the conclusions are more applicable to the American population. Existing studies have demonstrated that due to physical and psychological differences, the suicide rate among depressed men is higher than that of women, especially in middle-aged and elderly individuals (51). Additionally, cross-country racial differences are crucial factors influencing the prevalence of depression and the quality of treatment (52–54). Based on the above studies, we have taken into account the limitations of LAP and focused on Chinese middle-aged and elderly men to investigate the relationship between LAP and the prevalence of depression in this group, in order to summarize the characteristic manifestations of different countries and different study populations.

In this study, we observed that contrary to previous findings which indicated a positive correlation between LAP and the prevalence of depression, LAP exhibited a negative association among middle-aged and elderly Chinese men. This U-shaped relationship may be attributed to the heterogeneity of lipids and distinct biological mechanisms. Lipids encompass a diverse group of molecules, including esters and derivatives composed of fatty acids and alcohols, each with multifaceted effects on mental health. For instance, research has demonstrated that Omega-3 eicosapentaenoic acid is linked to enhanced wellbeing and achievement (55), and that fish oil supplements rich in omega-3 eicosapentaenoic acid are more effective than placebos in treating depressive symptoms (56). These observations suggest that lipid accumulation does not uniformly represent all lipids, and their impact on depression can be bidirectional. Therefore, we propose that the U-shaped association between LAP and depression may stem from the bidirectional mood regulation by lipids, involving neuroinflammation, hormonal modulation, lipid regulatory and protective effects, as well as the interplay between psychosocial factors and eating behaviors.

Previous studies have demonstrated that the accumulation of visceral fat induces chronic low-grade systemic inflammation via the secretion of inflammatory factors, such as tumor necrosis factor α, which can penetrate the blood-brain barrier and impact the emotion-regulating regions (44). Although excessive fat accumulation exacerbates depression by amplifying inflammatory responses, in cases of high-inflammation-induced depression, unsaturated fatty acids from specific high-fat foods (such as those rich in ω-3 fatty acids) can effectively combat neuroinflammation by modulating G-protein functions in lipid rafts and altering neurotransmitter signal transduction pathways, thereby alleviating depressive symptoms (57). Beyond their role in energy metabolism, dietary fatty acids serve as regulators of gene expression for enzymes and proteins involved in GCBC utilization (58), exerting a negative regulatory effect on inflammatory depression. Additionally, research on older women has shown that supplementation with omega-3 fatty acids combined with aerobic exercise improves lipid profiles and significantly reduces depressive symptoms in obese women. The study by Irandoost et al. further corroborated the positive impact of exercise and dietary interventions on mitigating depression symptoms in elderly women, suggesting that regulating lipid metabolism can effectively influence emotional management (59, 60). Collectively, these findings indicate that moderate fat accumulation can stabilize neurotransmitters through promoting fatty acid metabolism and countering neuroinflammation, thereby playing a crucial role in emotional regulation (56, 57, 61, 62).Based on the existing evidence, researchers recommend that one of the five key dietary strategies for preventing depression is to consume a high intake of foods rich in omega-3 and other polyunsaturated fatty acids (63).

At the same time, eating behavior is also closely related to the bidirectional regulation of lipid on mood. Different races and countries have different dietary habits and cultural traditions, which leads to different manifestations of the relationship between LAP and depression prevalence in different racial and national groups. Some studies have shown that among middle-aged black and white individuals, there is a correlation between higher optimism and lower levels of TC and LDL-C in each subgroup, and this correlation is stronger in white women (64); another study indicated that in the Chinese adult population, individuals with a higher optimism index tend to have a higher BMI (65). This may be because: in terms of ethnicity, different races have different body fat rates at the same lipid parameters, with Asians generally having a higher body fat rate than whites (66–72); in terms of subjective consciousness, Chinese people often derive pleasure from high-fat, high-calorie, high-protein foods; and in the relationship between taste and mood, individuals with mild depressive symptoms unconsciously consume more high-fat foods (73), but those with severe depression are more likely to develop anorexia. Simultaneously, numerous animal studies have demonstrated that neuropeptide Y (NPY), a 36-amino-acid peptide, exhibits both appetite-stimulating and antidepressant effects. This lipid-mediated dual regulatory mechanism of appetite and mood may also underlie the negative correlation observed between the LAP and depression (74).

Finally, physiological factors, social identity, psychological pressure, and emotional support also can modulate the impact of fat accumulation on depression. Moderate fat accumulation may offer both psychological and physiological benefits for some individuals, particularly middle-aged and older men. Existing studies have shown that women have more hormonal fluctuations and are more sensitive to hormonal changes than men (75), and the negative correlation between male abdominal obesity and depressive symptoms is stronger than in women (76). Additionally, the negative correlation between obesity indices and depressive symptoms intensifies with age, potentially due to differing psychosocial characteristics and self-perceptions of “obesity” among younger vs. middle-aged and older adults (28, 77).Young adults typically encounter greater societal pressure to maintain an attractive appearance. In the face of social stigmatization due to obesity, they often struggle to regulate their emotions effectively, leading to internalized self-esteem issues stemming from weight-related stigma, thereby increasing the risk of depression (78–80). Conversely, as individuals age, the emotional impact of body image diminishes in middle-aged and elderly populations. Psychological factors such as feelings of inferiority and perceptions of obesity may no longer elicit negative emotional responses in older adults (81). Unlike the “appearance anxiety” prevalent among younger individuals, middle-aged and elderly people tend to prioritize health functionality over physical appearance. Although their subjective health satisfaction may not always align with their actual health status, if an individual perceives their health positively, even in the presence of metabolic abnormalities, their risk of depression may be mitigated (65). This “buffer effect of health satisfaction” could partly explain the U-shaped relationship between LAP and depression—moderate lipid accumulation might be perceived as a metabolic reserve rather than a health threat in middle-aged and older adults. Additionally, studies have shown that abdominal obesity is associated with an increased risk of depression in individuals under 60 years old, while this association weakens in those over 60 (82). This may be attributed to the fact that self-esteem tends to stabilize in middle and old age, becoming less reliant on external validation, thereby reducing the negative impact of self-esteem on the correlation between obesity and depression.

Research on the relationship between obesity and depression is already a matter of much debate, and there is no clear conclusion to date. This is not only related to the diversity of lipids themselves but also to the complexity of depression and the characteristics of the study population. Some studies have shown that lipid parameters can affect cognition not only through direct biological pathways but also indirectly through psychological factors such as depression, leading to cognitive impairments (43). Other studies have shown that the specific knockout of fat mass and obesity-related proteins can induce depressive-like behaviors in mice, while the overexpression of fat mass and obesity-related proteins has antidepressant effects. These studies indicate that lipids and obesity exert bidirectional regulation on depression. Specifically, the interplay among lipid dysregulation, neuroinflammation, hormonal changes, and psychosocial factors likely contributes to the intricate relationship between LAP and depression. This highlights the potential role of biological pathways in mood regulation. Further investigation into these mechanisms can enhance our understanding of how lipid accumulation influences mood bidirectionally, thereby improving our ability to predict and prevent depression. At the same time, our study suggests that before reaching a threshold, a certain amount of lipid accumulation can significantly reduce the prevalence of depression in the Chinese middle-aged and elderly male population, but after reaching the threshold, excessive lipid accumulation will increase the prevalence of depression in this group. Therefore, we partially support the “jolly fat” hypothesis in the Chinese middle-aged and elderly male population, which is consistent with many previous studies (83, 84).

## 5 Strengths and limitations

This study is the first to discuss the relationship between LAP and the prevalence of depression among Chinese middle-aged and elderly men, and it also preliminarily explores the influencing factors of depression prevalence in this group, as well as the association between LAP and comorbid stroke and stroke-related depression. The study takes into account the limitations of LAP and the characteristics of different study populations, which can better enhance the representativeness of the conclusions. Secondly, the stability of the results and robustness of the model are effectively proved by subgroup analysis interaction test and sensitivity analysis. In addition, by adjusting for other confounding factors, a significant atypical U-shaped relationship between LAP and the prevalence of depression in the study population is further recognized. Then, by calculating the corresponding cut points of the sample data and determining the threshold values, the conclusions can be more clearly explained. Finally, the predictive performance of LAP, BMI, and other metabolic marker models regarding depression in participants was compared and analyzed. This analysis robustly demonstrated the significant potential of LAP for diagnosing and predicting depressive tendencies among middle-aged and elderly individuals in China. The study proposes hypotheses and viewpoints different from other similar studies, which can provide certain support for the “jolly fat” hypothesis and also offer a reference for future more refined stratified, racial, and population-based studies. However, the study still has certain limitations. Since the sample data of CHARLS is obtained by organizing the results of survey questionnaires, the answers filled in according to the participants' subjective wishes may lead to “false diagnosis” or “wrong diagnosis” of depressive symptoms, resulting in a mismatch between the number of cases included in the study and the actual number of cases, thus generating a certain error. Due to the cross-sectional nature of the study design, it is not feasible to establish causality. Consequently, while this study identifies a correlation between LAP levels and the prevalence of depression in middle-aged and elderly Chinese men, it cannot confirm whether higher or moderate LAP levels have a preventive effect on depression within this population. Additionally, it cannot determine whether lower LAP levels are a direct or indirect consequence of depression. Secondly, considering the overall prevalence of depression in men, more samples may still need to be included in the study. In addition, while the study incorporates several confounding factors to ensure comprehensiveness, it may still overlook other lifestyle-related confounders, potentially introducing bias into the results. Lastly, the conclusions of this study still lack a certain generalizability. Although CESD-based screening for depressive symptoms is widely validated, recall or response biases may still exist. Therefore, future studies should quantify the contribution of psychological variables and other factors to the study's conclusions using multidimensional scales (such as the Rosenberg Self-Esteem Scale, Body Satisfaction Questionnaire, and PHQ-9 Depression Screening Scale) to more accurately and objectively diagnose outcome variables. Moreover, this study should include larger and more diverse samples from different databases and conduct comparative analyses across various racial and gender groups to broaden the scope and enhance the depth and breadth of the research.

## 6 Conclusion

This study reveals for the first time a stable yet atypical U-shaped relationship between LAP and the prevalence of depression among middle-aged and elderly Chinese men, providing robust evidence for the “jolly fat” hypothesis. The effective threshold of LAP identified in this study indicates that within a specific range, an increase in LAP levels can significantly reduce the risk of depression in this demographic. Furthermore, the ROC curve and AUC value demonstrate that LAP is more effective in predicting depression in this group compared to traditional indices such as BMI and simple biological metabolites. This conclusion not only offers a new theoretical foundation for mental health management of middle-aged and elderly Chinese men but also provides critical insights for intervention measures and dietary guidance in public health. For instance, during periods without or with mild depression, controlled fat intake and lipid accumulation can effectively mitigate depressive symptoms and delay disease progression. From a clinical perspective, the threshold of 63.374 proposed in this study not only offers valuable reference for diagnosing and identifying patients at high risk of depression but also supports the development of appropriate treatment plans. Additionally, policymakers can utilize this threshold as a benchmark for establishing public health screening standards to enhance mental health support, particularly for individuals with obesity. However, the threshold values obtained in this study do not confirm their universality across different ethnicities, genders, or age groups. Therefore, further verification is required in diverse populations to quantify fat intake and determine the effective level of LAP. As an indicator of visceral fat measurement, LAP holds greater promise compared to traditional biomarkers and can be integrated with other clinical indicators to guide nutritional management for individuals with depression. Consequently, future investigations should focus on accounting for differences in factors such as ethnicity, gender, and lifestyle to strengthen the applicability of LAP and its threshold values across various populations to enhance large-scale screening and preventive measures in public health, ultimately contributing to improved health outcomes and quality of life for the community.

## Data Availability

The original contributions presented in the study are included in the article/Supplementary material, further inquiries can be directed to the corresponding author/s.
