# Supplementary material for: A significant atypical U-shaped relationship exists between Lipid Accumulation Product and depression prevalence among Chinese middle-aged and elderly men: a cross-sectional study based on CHARLS
Source: Front Nutr. 2025 May 21;12:1561990. doi: 10.3389/fnut.2025.1561990 (PMC12133478; doi:10.3389/fnut.2025.1561990)
Supplement: Supplementary file 1 [file Table_1.docx]

Supplementary Material

# Supplementary Figures and Tables

## Supplementary Figures


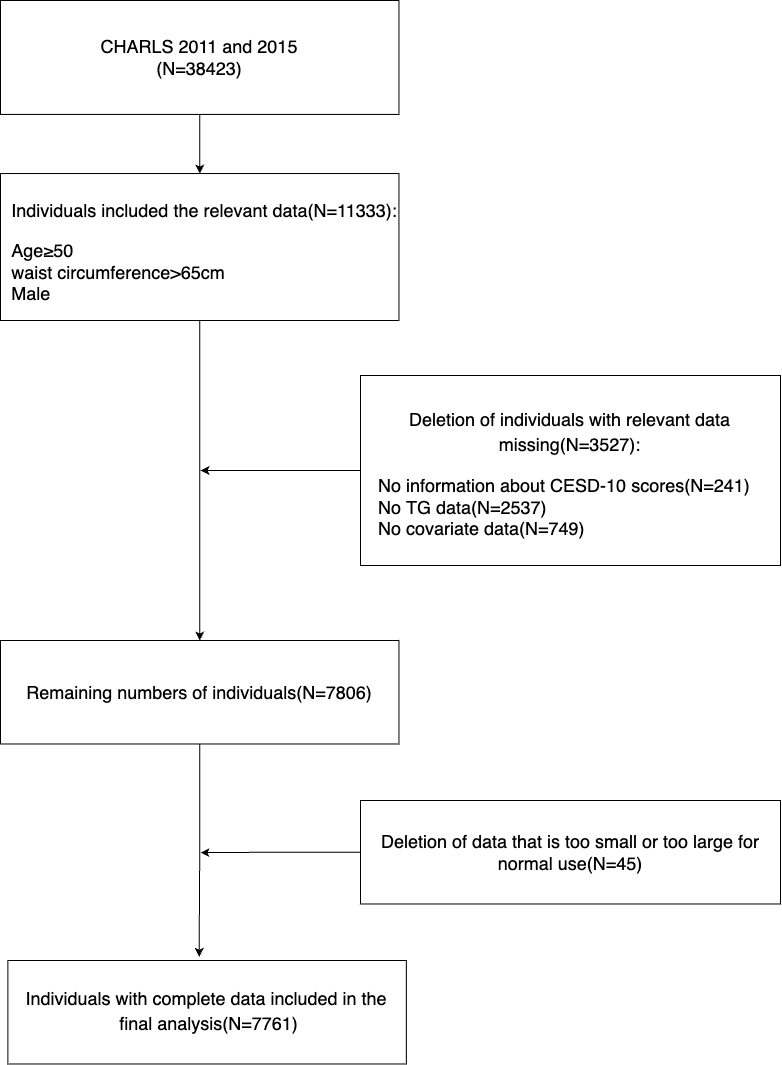


**Fig.1** The flow chart of the included participants in this study.

**Fig.2** RCS curve model of the association between LAP and depression prevalence among middle-aged and elderly Chinese men.


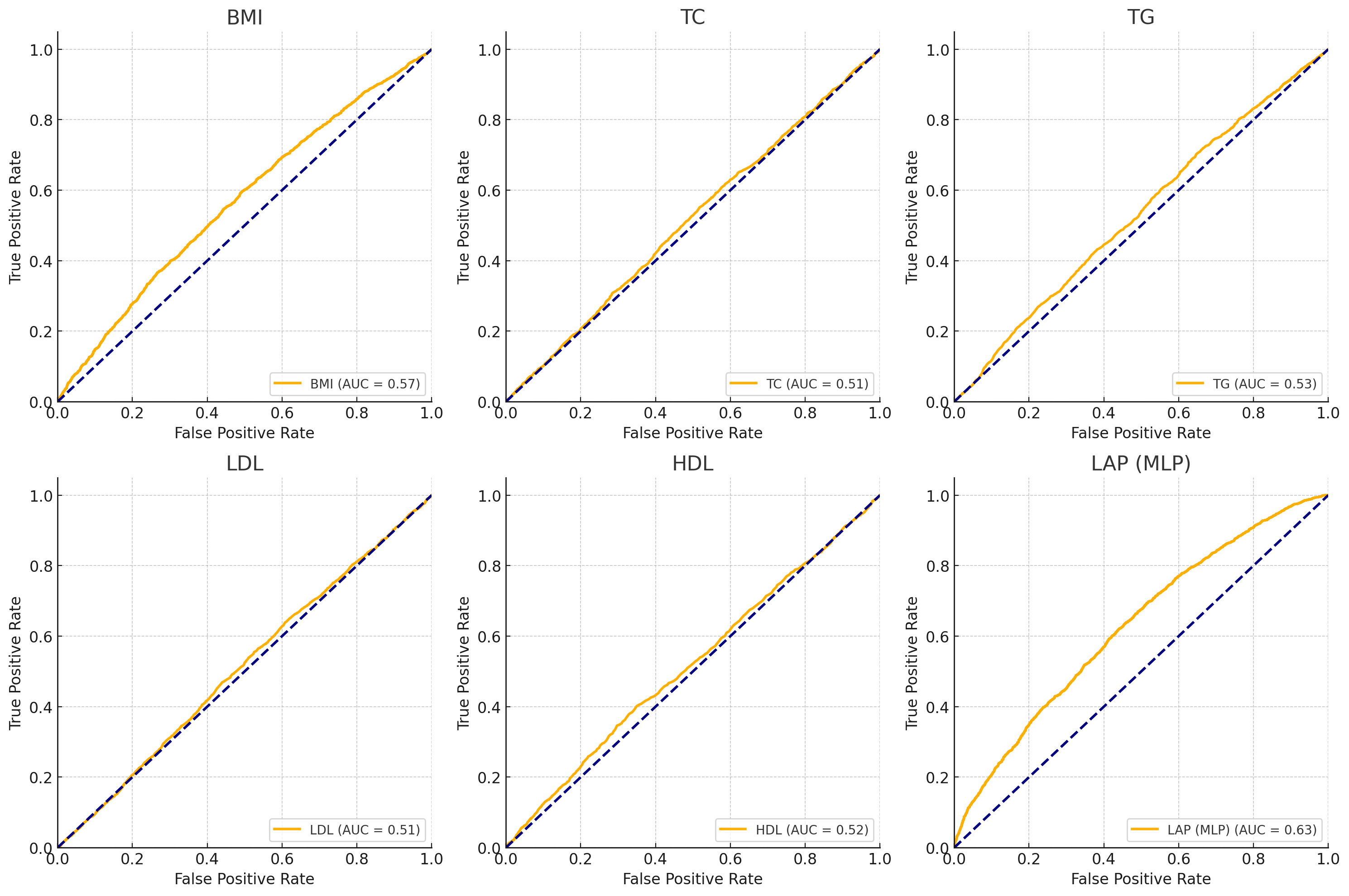


**Fig.3** ROC curves for predicting depression using LAP, BMI, and metabolic biomarkers.

## Supplementary Tables

Table 1

Characteristics of participants in the CHARLS 2011 and 2015 (n =7761).

|  | **lipid accumulation product（LAP）（N=7761）** | | | **P-value** |
| --- | --- | --- | --- | --- |
|  | **T1（N=2585）** | **T2（N=2589）** | **T3（N=2587）** |  |
| Age,years | 64.04 ± 8.50 | 63.43 ± 8.23 | 61.40 ± 7.74 | <0.001 |
| Married,% | 2268 (87.74%) | 2303 (88.95%) | 2393 (92.50%) | <0.001 |
| Educated,% | 1517 (58.68%) | 1717 (66.32%) | 1918 (74.14%) | <0.001 |
| Smoking,% | 1625 (62.86%) | 1421 (54.89%) | 1226 (47.39%) | <0.001 |
| Drinking,% | 1413 (54.66%) | 1437 (55.50%) | 1460 (56.44%) | 0.438 |
| Exercise,% | 1068 (90.20%) | 1099 (90.68%) | 1087 (89.91%) | 0.813 |
| ADL6,% | 417 (16.13%) | 440 (16.99%) | 433 (16.74%) | 0.693 |
| Height,m | 1.62 ± 0.08 | 1.63 ± 0.08 | 1.65 ± 0.07 | <0.001 |
| Weight,kg | 53.46 ± 7.27 | 61.93 ± 8.71 | 71.26 ± 10.64 | <0.001 |
| BMI,kg/m2 | 20.60 ± 7.59 | 23.46 ± 10.91 | 26.44 ± 17.02 | <0.001 |
| BMI,kg/m2(Tertile) |  |  |  | <0.001 |
| < 18.5 | 437 (16.91%) | 50 (1.93%) | 8 (0.31%) |  |
| 18.5 to <24 | 2030 (78.53%) | 1682 (64.97%) | 712 (27.52%) |  |
| ≥ 24 | 118 (4.56%) | 857 (33.10%) | 1867 (72.17%) |  |
| HTN,% | 1015 (39.26%) | 1345 (51.95%) | 1668 (64.48%) | <0.001 |
| DM,% | 269 (10.41%) | 346 (13.36%) | 689 (26.63%) | <0.001 |
| Dyslipidemia,% | 172 (6.65%) | 311 (12.01%) | 663 (25.63%) | <0.001 |
| Stroke,% | 72 (2.79%) | 88 (3.40%) | 125 (4.83%) | <0.001 |
| Psychosis,% | 37 (1.43%) | 32 (1.24%) | 28 (1.08%) | 0.527 |
| Metabolic biomarkers |  |  |  |  |
| Total cholesterol,mg/dl | 173.36 ± 33.36 | 180.80 ± 34.91 | 191.34 ± 38.04 | <0.001 |
| Triglycerides,mg/dl | 74.34 ± 28.85 | 103.96 ± 36.58 | 209.58 ± 114.79 | <0.001 |
| LDL cholesterol,mg/dl | 99.61 ± 29.19 | 108.33 ± 30.75 | 106.55 ± 34.57 | <0.001 |
| HDL cholesterol,mg/dl | 57.90 ± 15.16 | 51.04 ± 12.85 | 42.77 ± 10.08 | <0.001 |

Abbreviation: T: Tertile; LDL: Low-Density Lipoprotein Cholesterol; HDL: High-Density Lipoprotein Cholesterol; DM: Diabetes Mellitus; HTN: Hypertension; BMI: Body Mass Index.ADL6:Activities of Daily Living Scale.

Table 2

Baseline prevalence of depressive symptoms among participants.

| **Depressive symptoms (n, %)** | **lipid accumulation product（LAP）（N=7761）** | | | **P-value** |
| --- | --- | --- | --- | --- |
|  | **T1（N=2585）** | **T2（N=2589）** | **T3（N=2587）** |  |
| Overall depressive symptom | 882 (34.12%) | 713 (27.54%) | 637 (24.62%) | <0.001 |
| Mild depressive symptom | 476 (18.41%) | 409 (15.80%) | 382 (14.77%) | <0.001 |
| Medium depressive symptom | 372 (14.39%) | 272 (10.51%) | 231 (8.93%) | <0.001 |
| Severe depressive symptom | 34 (1.32%) | 32 (1.24%) | 24 (0.93%) | <0.001 |
| Stroke only | 29 (1.12%) | 55 (2.12%) | 68 (2.63%) | <0.001 |
| Depressive symptoms only | 839 (32.46%) | 680 (26.26%) | 580 (22.42%) | <0.001 |
| Stroke and Depressive symptoms | 43 (1.66%) | 33 (1.27%) | 57 (2.20%) | <0.001 |

Table 3

Univariate analysis of the association between depression with covariates.

| **Variable** | **OR (95 % CI)** | **P-value** |
| --- | --- | --- |
| Age | 1.01(1.00, 1.02) | 0.0005 |
| Married |  |  |
| No | Ref | - |
| Yes | 0.53 (0.46, 0.62) | <0.0001 |
| Educated |  |  |
| No | Ref | - |
| Yes | 0.61 (0.55, 0.68) | <0.0001 |
| Smoking |  |  |
| No | Ref | - |
| Yes | 1.18 (1.06, 1.30) | 0.0014 |
| Drinking |  |  |
| No | Ref | - |
| Yes | 0.77 (0.70, 0.85) | <0.0001 |
| Exercise |  |  |
| No | Ref | - |
| Yes | 0.87 (0.68, 1.10) | 0.2467 |
| ADL6 |  |  |
| No | Ref | - |
| Yes | 3.90 (3.45, 4.42) | <0.0001 |
| Height | 0.17 (0.09, 0.33) | <0.0001 |
| Weight | 0.98 (0.97, 0.98) | <0.0001 |
| BMI | 0.97 (0.96, 0.98) | <0.0001 |
| BMI (Tertile) |  |  |
| <18.5 | Ref | - |
| >=18.5, <24 | 0.71 (0.59, 0.86) | 0.0005 |
| >=24 | 0.49 (0.40, 0.60) | <0.0001 |
| HTN |  |  |
| No | Ref | - |
| Yes | 1.04 (0.94, 1.15) | 0.4053 |
| DM |  |  |
| No | Ref | - |
| Yes | 1.11 (0.98, 1.27) | 0.1078 |
| Dyslipidemia |  |  |
| No | Ref | - |
| Yes | 1.15 (1.01, 1.32) | 0.0377 |
| Stroke |  |  |
| No | Ref | - |
| Yes | 2.24 (1.77, 2.84) | <0.0001 |
| Psychosis |  |  |
| No | Ref | - |
| Yes | 3.03 (2.03, 4.54) | <0.0001 |

Abbreviation: DM: Diabetes Mellitus; HTN: Hypertension; BMI: Body Mass Index; CI, confidence interval; OR: odds ratio.ADL6:Activities of Daily Living Scale.

Table 4

Multivariate Regression Equations for LAP, Metabolic Biomarkers, and Depression

| **Exposure** | **Tertile group** | **Model 1** | | **Model 2** | | **Model 3** | |
| --- | --- | --- | --- | --- | --- | --- | --- |
|  |  | **OR(95% CI)** | **P-value** | **OR(95% CI)** | **P-value** | **OR(95% CI)** | **P-value** |
| LAP | Low | Ref | - | Ref | - | Ref | - |
|  | Middle | 0.73 (0.65, 0.83) | <0.0001 | 0.73 (0.61, 0.88) | 0.0009 | 0.73 (0.60, 0.88) | 0.0010 |
|  | High | 0.63 (0.56, 0.71) | <0.0001 | 0.65 (0.54, 0.79) | <0.0001 | 0.61 (0.49, 0.76) | <0.0001 |
| TC | Low | Ref | - | Ref | - | Ref | - |
|  | Middle | 0.94 (0.84, 1.06) | 0.3296 | 0.97 (0.80, 1.16) | 0.7271 | 0.97 (0.81, 1.17) | 0.7691 |
|  | High | 0.92 (0.81, 1.04) | 0.1665 | 0.91 (0.75, 1.09) | 0.3055 | 0.90 (0.74, 1.09) | 0.2638 |
| TG | Low | Ref | - | Ref | - | Ref | - |
|  | Middle | 0.93 (0.83, 1.05) | 0.2365 | 0.96 (0.80, 1.15) | 0.6407 | 0.98 (0.81, 1.18) | 0.7952 |
|  | High | 0.75 (0.66, 0.85) | <0.0001 | 0.83 (0.68, 1.00) | 0.0511 | 0.83 (0.68, 1.01) | 0.0691 |
| LDL | Low | Ref | - | Ref | - | Ref | - |
|  | Middle | 0.99 (0.88, 1.11) | 0.8432 | 1.00 (0.84, 1.20) | 0.9732 | 1.02 (0.85, 1.22) | 0.8647 |
|  | High | 0.90 (0.79, 1.01) | 0.0761 | 0.79 (0.66, 0.96) | 0.0179 | 0.80 (0.66, 0.97) | 0.0221 |
| HDL | Low | Ref | - | Ref | - | Ref | - |
|  | Middle | 0.97 (0.86, 1.10) | 0.6781 | 0.99 (0.82, 1.20) | 0.8977 | 0.97 (0.80, 1.18) | 0.7780 |
|  | High | 1.21 (1.08, 1.37) | 0.0017 | 1.32 (1.09, 1.59) | 0.0048 | 1.30 (1.06, 1.59) | 0.0105 |

Abbreviation: TC: Total Cholesterol; TG: Triglycerides; LDL: Low-Density Lipoprotein Cholesterol; HDL: High-Density Lipoprotein Cholesterol; CI: confidence interval; OR: odds ratio.

model 1: Non-adjusted

model 2: adjust for Age; Married; Educated; Smoking; Drinking; Exercise; ADL6

model 3: adjust for Age; Married; Educated; Smoking; Drinking; Exercise; ADL6; BMI; HTN; DM; Dyslipidemia; Stroke; Psychosis

Table 5

Hierarchical analysis and interaction effects.

| **Variables^a^** | **lipid accumulation product（LAP）** | | | | | **P for**  **interaction** |
| --- | --- | --- | --- | --- | --- | --- |
|  | **TI** | **T2** | **P-value** | **T3** | **P-value** |  |
| Age(Tertile) |  |  |  |  |  | 0.1509 |
| 50 - 58 | Ref | 0.68 (0.55, 0.86) | 0.0009 | 0.64 (0.52, 0.80) | <0.0001 |  |
| 59 - 65 | Ref | 0.79 (0.64, 0.98) | 0.0282 | 0.59 (0.48, 0.73) | <0.0001 |  |
| 66 - 94 | Ref | 0.73 (0.61, 0.88) | 0.0012 | 0.69 (0.56, 0.85) | 0.0004 |  |
| Married |  |  |  |  |  | 0.0264 |
| No | Ref | 1.03 (0.74, 1.42) | 0.8683 | 0.90 (0.63, 1.30) | 0.5821 |  |
| Yes | Ref | 0.70 (0.62, 0.80) | <0.0001 | 0.62 (0.54, 0.71) | <0.0001 |  |
| Smoking |  |  |  |  |  | 0.0061 |
| No | Ref | 0.73 (0.61, 0.89) | 0.0013 | 0.67 (0.56, 0.81) | <0.0001 |  |
| Yes | Ref | 0.75 (0.64, 0.87) | 0.0002 | 0.62 (0.52, 0.73) | <0.0001 |  |
| Drinking |  |  |  |  |  | 0.1325 |
| No | Ref | 0.73 (0.61, 0.87) | 0.0004 | 0.67 (0.56, 0.80) | <0.0001 |  |
| Yes | Ref | 0.74 (0.63, 0.87) | 0.0003 | 0.60 (0.51, 0.71) | <0.0001 |  |
| Exercise |  |  |  |  |  | 0.9375 |
| No | Ref | 0.78 (0.41, 1.51) | 0.4664 | 0.55 (0.24, 1.27) | 0.1615 |  |
| Yes | Ref | 0.77 (0.64, 0.94) | 0.0087 | 0.70 (0.57, 0.87) | 0.0014 |  |
| ADL6 |  |  |  |  |  | 0.0165 |
| No | Ref | 0.76 (0.66, 0.88) | 0.0002 | 0.68 (0.58, 0.80) | <0.0001 |  |
| Yes | Ref | 0.63 (0.48, 0.84) | 0.0019 | 0.45 (0.32, 0.62) | <0.0001 |  |
| BMI(Tertile) |  |  |  |  |  | 0.1414 |
| <18.5 | Ref | 0.60 (0.31, 1.14) | 0.1204 | 2.57(0.61, 10.88) | 0.2006 |  |
| >=18.5, <24 | Ref | 0.87 (0.76, 1.00) | 0.0559 | 0.76 (0.63, 0.92) | 0.0048 |  |
| >=24 | Ref | 0.55 (0.36, 0.83) | 0.0043 | 0.58 (0.39, 0.85) | 0.0060 |  |
| HTN |  |  |  |  |  | 0.4227 |
| No | Ref | 0.74 (0.62, 0.87) | 0.0002 | 0.57 (0.47, 0.69) | <0.0001 |  |
| Yes | Ref | 0.71(0.60,0.85) | 0.0002 | 0.64 (0.54, 0.75) | <0.0001 |  |
| DM |  |  |  |  |  | 0.3975 |
| No | Ref | 0.75 (0.66, 0.85) | <0.0001 | 0.60 (0.53, 0.69) | <0.0001 |  |
| Yes | Ref | 0.61 (0.43, 0.85) | 0.0039 | 0.61 (0.45, 0.82) | 0.0009 |  |
| Dyslipidemia |  |  |  |  |  | 0.0055 |
| No | Ref | 0.71 (0.63, 0.81) | <0.0001 | 0.62 (0.55, 0.71) | <0.0001 |  |
| Yes | Ref | 0.74 (0.51, 1.09) | 0.1255 | 0.49 (0.35, 0.69) | <0.0001 |  |
| Stroke |  |  |  |  |  | 0.6509 |
| No | Ref | 0.75 (0.66, 0.84) | <0.0001 | 0.61 (0.54, 0.70) | <0.0001 |  |
| Yes | Ref | 0.40 (0.21, 0.77) | 0.0055 | 0.57 (0.31, 1.02) | 0.0573 |  |
| Psychosis |  |  |  |  |  | 0.0038 |
| No | Ref | 0.74 (0.66, 0.83) | <0.0001 | 0.63 (0.56, 0.71) | <0.0001 |  |
| Yes | Ref | 0.53 (0.20, 1.38) | 0.1946 | 1.05 (0.39, 2.87) | 0.9186 |  |
| TC |  |  |  |  |  | 0.6802 |
| Low | Ref | 0.88 (0.73, 1.07) | 0.2084 | 0.71 (0.57, 0.89) | 0.0024 |  |
| Middle | Ref | 0.64 (0.52, 0.78) | <0.0001 | 0.49 (0.40, 0.61) | <0.0001 |  |
| High | Ref | 0.68 (0.55, 0.86) | 0.0010 | 0.69 (0.56, 0.86) | 0.0007 |  |
| LDL(Tertile) |  |  |  |  |  | 0.7340 |
| Low | Ref | 0.78 (0.63, 0.96) | 0.0168 | 0.61 (0.49, 0.74) | <0.0001 |  |
| Middle | Ref | 0.81 (0.66, 0.98) | 0.0344 | 0.65 (0.52, 0.80) | <0.0001 |  |
| High | Ref | 0.64 (0.52, 0.80) | <0.0001 | 0.64 (0.52, 0.79) | <0.0001 |  |
| HDL(Tertile) |  |  |  |  |  | 0.6665 |
| Low | Ref | 0.66 (0.50, 0.86) | 0.0020 | 0.63 (0.49, 0.80) | 0.0002 |  |
| Middle | Ref | 0.82 (0.67, 1.01) | 0.0563 | 0.62 (0.49, 0.77) | <0.0001 |  |
| High | Ref | 0.73 (0.61, 0.88) | 0.0008 | 0.63 (0.47, 0.83 | 0.0013 |  |

Abbreviation: T: Tertile, LDL: Low-Density Lipoprotein Cholesterol; HDL: High-Density Lipoprotein Cholesterol; DM: Diabetes Mellitus; HTN: Hypertension; BMI: Body Mass Index; TC: Total Cholesterol.ADL6:Activities of Daily Living Scale.

^a^ All variables were adjusted for each element and analyzed.

Table 6

Sensitivity analysis result.

| **Variables** | **Coefficient** | **Intercept** |
| --- | --- | --- |
| Married |  |  |
| No | -0.00295 | -0.2759 |
| Yes | -0.00383 | -0.8507 |
| Smoking |  |  |
| No | -0.00261 | -0.9017 |
| Yes | -0.00540 | -0.6782 |
| Dyslipidemia |  |  |
| No | -0.00512 | -0.7813 |
| Yes | -0.00440 | -0.5522 |
| Psychosis |  |  |
| No | -0.00455 | -0.7761 |
| Yes | 0.00545 | -0.0102 |

**Coefficient：regression coefficient.**
